# Clonal analysis of palmar fibromatosis: a study whether palmar fibromatosis is a real tumor

**DOI:** 10.1186/1479-5876-4-21

**Published:** 2006-05-12

**Authors:** Lei Wang, Hongguang Zhu

**Affiliations:** 1 Department of Pathology, Fudan University Shanghai Medical College, Yixueyuan Road 138, Shanghai, China

## Abstract

**Background:**

Palmar fibromatosis that arises in the palmar soft tissue is characterized by infiltrative growth with a tendency toward local recurrence but does not metastasize. This study investigated the clonality of this process in twelve female patients, each with a single lesion, by examining the pattern of X-chromosome inactivation.

**Methods:**

Hematoxylin and eosin stained sections of formalin-fixed, paraffin-embedded tissues were microdissected by laser capture microdissection to obtain the proliferative spindle cells. Tumor cells were isolated from the sections of rectum adenocarcinoma, and used for positive control. The genomic DNAs was extracted with phenol-chloroform, digested with a methylation-sensitive restriction endonuclease HpaII, and amplified by polymerase chain reaction (PCR), using primers targeted to a highly polymorphic short tandem repeat (STR) of the human androgen receptor gene (HUMARA).

**Results:**

Among the twelve samples, three samples failed amplification, one sample showed homozygosity which was not suitable for further analysis, eight samples were successfully amplified, and showed a random X chromosome inactivation pattern, suggesting polyclonality of these lesions.

**Conclusion:**

The current findings suggest that palmar fibromatosis is a reactive proliferation rather than a clonal neoplasm.

## Background

Fibromatosis is a group of proliferative disorders of soft tissue characterized by infiltrative pattern of growth with repeated local recurrences, and proliferation of uniform well-differentiated spindled cells (mainly myofibroblasts) with presence of a variable amount of collagen between the proliferating cells. Although the lesions of fibromatosis are often locally aggressive, they lack the capacity of metastasis. [1,2]. Palmar fibromatosis, also known as Dupuytren's disease, is one kind of superficial fibromatosis. There are three distinct histological phases during the ontogeny described by Luck [3]. The proliferative phase is characterized by nodular lesion with a proliferation of myofibroblasts which express α-smooth muscle actin (α-SMA). Mitotic figures are usually infrequent, but in this phase locally prominent mitotic figures may be observed [1]. In evolutional stage, a majority of myofibroblasts are substituted by fibroblasts and spindled cells were separated by the collagen. And in the residual phase, the nodule is replaced by scar-like tissue and therefore, no expression of α-SMA due to the diminishing of myofibroblasts.

The pathogenesis of fibromatosis is poorly understood. Whether fibromatosis are reactive or neoplastic lesions has long been controversial. One of the essential tenets in defining a neoplastic proliferation is that the cells are originated from a single clone [4,5]. In contrast, normal tissue and reactive proliferation are polyclonal. Several studies [6-8] indicate that desmoid fibromatosis, a subtype of fibromatosis reside in the deep soft tissue, is a true type of neoplastic process instead of a polyclonal reactive proliferation. Chansky [9] assessed the clonality of palmar fibromatosis using lesional tissue from 2 patients and the result showed that the tissues from both patients are polyclonal. However, additional cases are needed to conclude that palmar fibromatosis is reactive proliferation process. In our study, tissues from 12 female patients with palmar fibromatosis were collected and the methylation inactivation pattern on X-chromosome were evaluated to determine clonality of palmar fibromatosis.

According to the Lyon hypothesis [10,11], one of the two X-chromosomes in each cell is inactivated by hypermethylation during the process of embryogenesis in females and the methylation patterns are highly conserved in subsequent somatic-cell divisions. Normal tissues from women consist cells randomly carry equal frequency of paternal and maternal methylated X-chromosome and therefore, are composed of a mosaic type in methylation patterns due to the random inactivation by methylation. In contrast, each individual cell in a clone derived from a common progenitor, maintains the same sequence methylation patterns of X-chromosome inactivation and the same allele is exclusively methylated. Methylation-sensitive restriction digestion followed by Polymerase chain reaction (PCR)-based methods are used to analyze the pattern of X-chromosome inactivation. The results are informative to tissues from only female patients who are heterozygous for a defined X-linked marker gene and carry approximately balanced methylation pattern for the given allele in normal condition. The methylation-sensitive restriction endonucleases HhaI or HpaII selectively target the unmethylated gene region derived from X-chromosome. In situation of balanced random methylation from normal tissue, both alleles of the marker gene are partial insensitive to the restriction digestion and therefore, both could be amplified utilizing flanking marker gene specific primer set under PCR reaction. On the contrary, marker gene from the same progenitor, inheriting the identical methylation patterns, only the methylated allele is insensitive to the enzyme cut and therefore, could be amplified by PCR while the other unmethylated allele could not be amplified due to the sensitivity to the enzyme. We investigated the clonality of palmar fibromatosis using the X-linked human androgen-receptor gene (HUMARA) assay. HUMARA is characterized by highly polymorphic trinucleotide-repeat (CAG) sequence proximal to methylation site with high incidence of heterozygosity [12], and the target gene amplicon is less than 300 bp. Those distinctiveness of the approach make it applicable to archival formalin-fixed tissues, which are often not amenable to analysis by a variety of alternative approaches [13].

## Methods

### Tissue samples

Formalin-fixed, paraffin-embedded surgical tissues were supplied by the department of pathology of Shanghai Huashan Hospital, Fudan University. Samples were selected from 12 female patients with palmar fibromatosis. One rectum adenocarcinoma specimen from a female patient was used as positive control. The clinicopathological features of the selected sample were summarized in Table 1. The mean age of the patients was 41.8 years (range 8–71). Each patient had a single lesion. Isolated firm palmar nodules from the palmar aponeurosis were used for the study. All cases were diagnosed by two experienced pathologists.

**Table 1:** Clinicopathological features and histopathological phases

*Case No*.	*Age(years)*	*Site*	*Size(cm)*	*Stage*
1	16	MP of index finger(L)	0.5 × 1.0	P.P. and E.P.
2	34	palmar surface(L)	1.0 × 2.0	P.P.
3	8	palmar surface(L)	1.0 × 1.5	P.P.
4	52	palmar surface(R)	1.5 × 1.5	P.P. and E.P.
5	25	MP of index finger(L)	4.0 × 4.0	P.P.
6	64	palmar surface(R)	0.3 × 0.3	E.P.
7	23	palmar surface(R)	1.0 × 1.5	E.P.
8	52	MP of middle finger(R)	0.3 × 0.5	P.P
9	42	MP of index finger(L)	0.5 × 1.0	P.P. and E.P.
10	71	palmar surface(R)	0.8 × 2.0	R.P.
11	62	palmar surface(R)	0.5 × 2.0	R.P.
12	52	MP of ring finger(R)	1.5 × 2.0	P.P.

MP = metacarpophalangeal joint; R = right hand; L = left hand;

P.P. = proliferative phase; E.P. = evolutional phase; R.P. = residual phase;

P.P. and E.P. = both proliferative phase and evolutional phase can be found in the same sample.

### Immunohistochemisitry

Immunohistochemical staining was performed on formalin-fixed paraffin-embedded sections, using the avidin-biotin-peroxidase complex (ABC) technique. Nonspecific binding sites were blocked with normal horse serum. The primary monoclonal antibody used in this study (1:100 diluted) was anti-α-smooth muscle actin (Cat.No. M-0004, Antibody Diagnostica Inc. U.S.).

### Microdissection and DNA extraction

**Figure 1:**
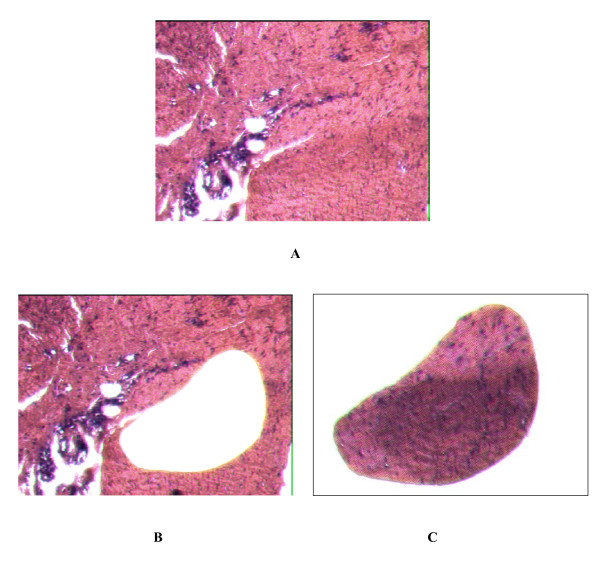
Samples of palmar fibromatosis before and after laser capture microdissection. Panel A (Hematoxylin eosin × 200) shows a section of palmar fibromatosis nodule from patient 9, with proliferative spindled cells. Panel B (Hematoxylin eosin × 200) shows the area with proliferative spindled cells which are removed by laser capture microdissection. Panel C the captured spindled cells.

We cut fifteen 10 μm thick sections from each block, and the sections were mounted on a 1.4 μm membrane with metal frame slides. The sections were dried for 3 hours at 55°C in incubator. Paraffin were removed in 2 steps xylene and the section were rehydrated in descending concentration of alcohol solutions sequentially. The section samples were stained with haematoxylin and eosin. Laser cut microdissection was performed with SL μCUT Laser Cut Microdissection (LCM) System (Molecular Machines and Industries, Switzerland). Spindled cells from palmar fibromatosis were captured on the adhesive caps (Fig. 1). Tumor cells isolated from the sections of rectum adenocarcinoma by LCM were used for positive control. After microdissection, the captured section on the caps were lysised in the present of 50 μl of proteinase K lysis buffer (500 μg/ml proteinase K, 0.1 M EDTA, 1% Tween-20, and Tris-HCl, PH 8.0) and digested at 55°C over night within the tube in inverted position. After incubation, the tubes were centrifuged for 5 minutes slowly and heat at 95°C for 10 minutes to inactivate the proteinase K. The DNA was extracted with phenol-chloroform and precipitated with isopropanol at -70°C for 1 hour. After 2 washes with 75% ethanol, isolated DNA were resuspended in 10 ul DEPC treated water.

### Enzyme digestion

One hundred to one hundred fifty nanogram DNA template was digested with 10 U (1 μl) of methylation-sensitive restriction endonuclease HpaII in the presence of 2 μl of 10×-REACT 8 enzyme buffer (Cat.No. 15209-018 GIBCO, U.S.) and 12 ul DEPC treated water. The test samples DNA were digested in identical fashion as the positive control utilizing rectum adenocarcinoma sample while the negative controls for each test and control experiments were carried in identical condition with the exception of the enzyme. Samples were incubated overnight at 37°C followed by inactivation at 95°C for 5 minutes.

### PCR amplification of human androgen receptor gene

Human androgen gene (HUMARA) is highly polymorphic characterized by variable trinucleotide-repeat ((CAG)_n _repeat n = 11–30) sequence in the first exon. The methylation-sensitive endonuclease HpaII recognition site locates upstream to the (CAG)_n _repeat 10. We designed the primers (5'-GCT GTG AAG GTT GCT GTT CCT CAT-3' and 5'-TCC AGA ATC TGT TCC AGA GCG TGC-3') flanking the HpaII site and short tandem repeat (STR) of CAG region to amply both alleles with different size of amplicons in natural condition and only the unmethylated allele after HpaII digestion.

PCR was performed in total volume of 20 μl of reaction mixture consisting 3 μl of digested or undigested DNA sample, 2 μl of 10 × reaction buffer, 1.6 μl of 25 mM MgCl_2_, 2 μl of dNTP (2 mM each of dATP, dTTP, dCTP, and dGTP), 1 μl each of primer (10 μM each), 0.3 μl of Taq polymerase (5 U/μl, SAGON, China), and 9.1 μl of distilled water. The PCR reaction was denatured at 95°C for 5 minutes and forty-five cycled at 95°C for 45 seconds, 63°C for 45 seconds and 72°C for 30 seconds with final extension at 72°C for 10 minutes. The PCR products were visualized first on 2% agarose gel to identify 250 bp–500 bp products which were then electrophoresised onto the 8 percent polyacrylamide gel. After stained with ethidium bromide, the gel were analysed with GeneTool Analysis Software (Syngene, U.K.) from which, a serial of intensity data of the PCR product bands were generated. In informative patients, two major HUMARA bands are expected with almost equal intensity, however, after methylation-sensitive endonuclease HpaII digestion, only one major band or marked reduction (more than 75 percent) in intensity of one band should be seen if the tissues were monoclonality [14].

## Results

### Histopathological and immunohistochemical features

**Figure 2:**
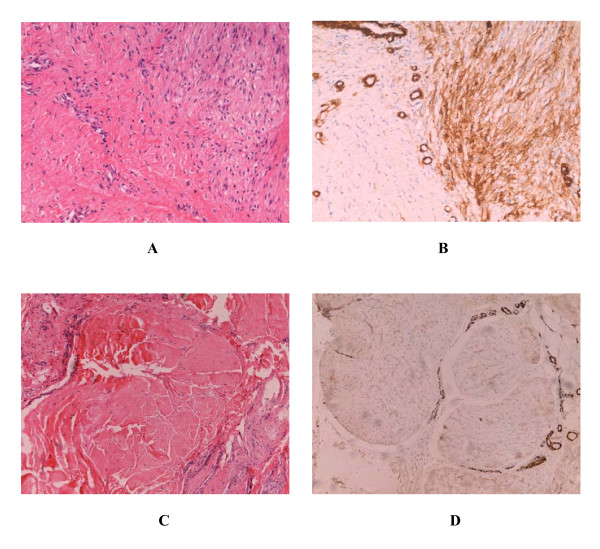
Histopathological and immunohistochemical features of palmar fibromatosis. Three distinct histological phases can be observed in the patient 9. The right part of Panel A (Hematoxylin and eosin × 200) shows proliferative phase of the lesion and the left part shows evolutional phase. Panel B (ABC × 200) shows the expression of α-SMA. Spindled cells of proliferative stage formed nodule and strongly expressed α-SMA. In evolutional stage, a majority of myofibroblasts were replaced by fibroblasts, and spindled cells were separated by the collagen. Panel C (Hematoxylin ane eosin × 200) shows residual phase of the lesion, and panel D (ABC × 200) shows the expression of α-SMA. Spindled cells disappear and are substituted by amounts of dense collagen. Except smooth muscle cells of blood vessels, α-SMA is negative.

All of the cases in our study had the classic histopathological and immunohistochemical feature of palmar fibromatosis. The proliferative phase was notably hypercellular. Spindled cells (mainly myofibroblast) that had uniform appearance formed nodules, and strongly expressed α-smooth muscle actin (α-SMA). With the development of the lesions (evolutional phase), the spindled cells arranged into lines and were separated by collagen (Fig. 2A and 2B). Older lesions were considerably less cellular, contained increased amounts of dense collagen, and were α-SMA-negative (Fig. 2C and 2D).

### Clonality analysis

**Figure 3:**
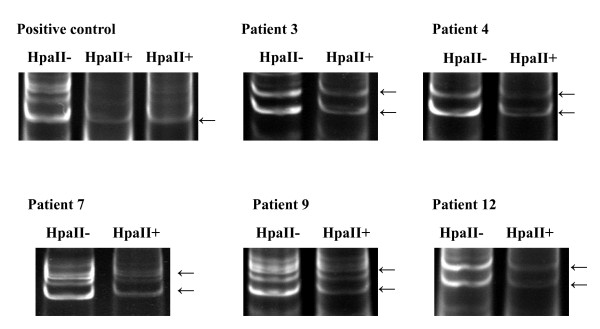
Clonal analysis by the pattern of X-chromosome inactivation (representative cases: cases 3, 4, 7, 9, 12, and positive control). The presence of multiple bands of each allele in the positive control, case 7 and case 9 is attributable to the slippage of DNA polymerase during amplification. The DNA undigested with methylation-sensitive endonuclease HpaII (HpaII-) produced two primary bands with equal intensity. After HpaII digestion (HpaII+), only a single band was observed in the sample of rectum adenocarcinoma used as positive control (arrow), in contrast, all samples of palmar fibromatosis presented two primary allelic bands (arrow), and there is no distinct reduction in the intensity of one of the alleles analyzed by GeneTool Analysis Software. Each of the eight patients had random inactivation of X-chromosome, which indicated palmar fibromatosis is polyclonal.

Three of the twelve patients (cases 5, 10, and 11) DNA sample failed to amplify expected PCR products visible on 2% agarose gel. Further analysis on 8% polyacrylamide gel demonstrate that multiple bands had been generated in some cases which could be the result of the slippage of DNA polymerase during amplification (Fig. 3) [14]. To by pass ambiguity, only dominate bands were chosen for further analysis. Among the samples succeeded in amplification, one patient (case 6) showed only one band even before restriction endonuclease digestion suggesting that the patient was homozygous at the HUMARA locus and was excluded for further study. The remaining eight patients and the control rectum adenocarcinoma patient were heterozygous indicated by two allelic bands of PCR products with approximately equal signal intensity and suitable for the further analysis. After enzymatic digestion, only a single band was observed in the sample of rectum adenocarcinoma used as positive control, validating that rectum adenocarcinoma was monoclonal and the clonal analysis method used in our study was viable (Fig. 3). In contrast, all eight samples of palmar fibromatosis presented exclusively two allelic bands after digestion. Quantitative analysis of PCR product intensity by the GeneTool Analysis Software (Syngene, U.K.) did not reveal distinct reduction in the intensity of either alleles, indicating that the inactivation of X-chromosome was random and the lesions were polyclonal in all eight patients (Fig. 3). These results suggested that palmar fibromatosis is a reactive proliferation rather than a clonal neoplasm.

## Discussion

The pathogenesis of palmar fibromatosis remains uncertain. The disease contains two fibrotic structures, the nodule and the cord. As the disease progresses, the nodule, which contains proliferative fibroblasts, with a high proportion being myofibroblasts, develops into the cord, a collagen-rich and acellular structure [15,16]. The appearance and disappearance of myofibroblasts in the lesions are similar to those observed in other tissues in which fibroblasts are present, such as granulation tissue. Interleukin-1(IL-1), platelet-derived growth factor-BB (PDGF-BB), and transforming growth factor-β (TGF-β) stimulate the growth of fibroblasts [17,18]. The effect of TGF-β on the proliferation of myofibroblasts has been studied in vitro [16]. The results indicate that TGF-β could induce fibroblast proliferation and increases the expression of α-SMA.

Desmoid fibromatosis (aggressive fibromatosis) is clonal fibroblastic proliferation. Despite the lack of the ability to metastasize, local recurrence is frequent and some desmoids prove fatal due to the madly local recurrence, especially in the significant viscera. In contrast, the rate of local recurrence of palmar fibromatosis is much lower, and the process of involution, from a cellular proliferation of nodule to the acellular scar-like tissue, is also different from desmoid fibromatosis.

The present study of fibromatosis shows that clonal chromosome changes are another feature of the disease process. Wever [19] found that the frequency of cytogenetic abnormalities varied with the type of fibromatosis. The results demonstrated that more than a half of samples from desmoid fibromatosis had clonal chromosome aberrations, and only 3 out of 28 specimens of superficial fibromatosis had aberrations. The frequent finding of clonal chromosome changes in desmoid fibromatosis confirms the neoplastic nature of demoid fibromatosis, which is in line with molecular data showing that demoid fibromatosis is monoclonal [6-8]. Our results suggested that palmar fibromatosis is a polyclonal reactive proliferation. It explains the infrequent chromosome aberrations in superficial fibromatosis, and the different clinic outcome and prognosis between palmar fibromatosis and desmoid fibromatosis. The both lesions belong to subtypes of fibromatosis, however, they are hyperplasia with completely different characters, namely one is reactive proliferation while the other is monoclonal proliferation.

Although the technique of clonality analysis based on the pattern of X-chromosome inactivation is powerful, there are still certain limitations. First, the purity of the cells we investigated could be reduced by the contamination of the normal cells, such as endothelial cells of vasculatures and fatty cells. Hence, it is unclear whether the appearance of polyclonality is genuine or due to contamination. In order to minimize any possibility of contamination, we used laser cut microdissection to gain the spindled cells, which allowed for rapid and accurate acquisition of cells that we were interested in [20-22], and tried to reduce the contamination of endothelial cells and fatty cells. Only a single band was observed in the sample of rectum adenocarcinoma, which was used as positive control. The result suggested that tumor cells are accurately harvested, none normal tissue components is existed, and the amount of methylation-sensitive restriction endonuclease we used is suitable, by which DNA template was completely digested. The test samples DNA were digested in identical fashion as the positive control. Second, being based on the pattern of X-chromosome inactivation, the technique of clonal analysis is only applied to female patients. The application of the lesions that are more common in men than women is limited, such as palmar fibromatosis. The third limitation is the occurrence of nonrandom X-chromosome inactivation (also known as skewing or unequal Lyonization) in healthy females. The skewed X-chromosome inactivation pattern mimics clonal derivation of cells, and makes clonality results non-informative [23]. In our study, eight informative samples were successful in amplification after digestion with methylation-sensitive restriction endonuclease HpaII, and presented two allelic bands of approximately equal intensity. The results not only assess palmar fibromatosis is polyclonality, but also show that none of the eight patients is the skewed X-chromosome inactivation pattern.

DNA from both frozen tissues and formalin-fixed, paraffin-embedded tissues were suitable for clonality analysis [24,25]. In the current study, only formalin-fixed, paraffin-embedded tissues due to the rare of palmar fibromatosis in women and the limited number of specimens suitable for analysis. This approach take the advantage of the large archival paraffin-embedded specimens that spanned many years and are easy to collect. In addition, the structure of tissues and the shapes of cells on paraffin-embedded specimens were clearer than frozen tissue, which made the diagnosis more credible and isolation of spindle cells of interest more precise. However, partial DNA template were degraded and damaged during process of formalin-fixed and paraffin-embedded, therefore, relative large amounts of DNA are needed from paraffin-embedded tissues comparing with frozen tissues. In our study, we cut fifteen 10 μm thick sections from each block, which ensured the amounts of DNA template, and we extracted DNA three times with Tris-buffer-saturated phenol-chloroform and once with chloroform, which purified crude DNA samples to ensure successful PCR amplification. Nevertheless, three of the twelve samples (cases 5, 10, and 11) fail to be amplified. We analyze the possible reasons for this failure. Both of lesions from patient 10 and patient 11 were in the residual stage, which was characterized by less cellular and increased amounts of dense collagen, therefore, there were so limited amounts of DNA template that PCR failed to amply. Surely, the damage of DNA template was not excluded. However, case 5 was in the proliferative phase, we speculated that there was scant optimal DNA template, which made a failure of PCR amplification. Because of the rupture of DNA, the PCR amplicon should be controlled within less than 500 base pairs. In our study, the DNA sequence we chosen to amplified was less than 300 base pairs, which ensured the success of PCR.

## Conclusion

In our study, we applied the analysis of the pattern of X-chromosome inactivation to determine the clonality of palmar fibromatosis. We discover that palmar fibromatosis is reactive proliferation process, which explained the different clinic outcome and prognosis between palmar fibromatosis and desmoid fibromatosis.

## Competing interests

The author(s) declare that they have no competing interests.

## Authors' contributions

W.L participated in the design of the study and carried out tissue samples collection, immunohistochemisitry, microdissection, DNA extraction, PCR, and completed the preparation of the manuscript. Z.H.G conceived of the study, participate in its design and coordination, and helped draft the manuscript.

## References

[B1] Fletcher CDM, Unni KK, Mertens F (2001). Pathology and Genetics of Tumours of Soft Tissue and Bone, World Health Organization Classification of Tumours (ed 1).

[B2] Juan Rosai (1996). Rosai and Ackerman's Surgical Pathology (ed 8).

[B3] Luck JV (1959). Dupuytren's contracture: a new concept of the pathogenesis correlated with surgical management. J Bone Joint Surg Am.

[B4] Nowell PC (1976). The clonal evolution of tumor cell populations. Science.

[B5] Shroyer KR, Gudlaugsson EG (1994). Analysis of clonality in archival tissue by polymerase chain reaction amplification of PGK-1. Hum Pathol.

[B6] Middleton SB, Frayling IM, Phillips RK (2000). Desmoids in familial adenomatous polyposis are monoclonal proliferations. Br J Cancer.

[B7] Li M, Cordon-Cardo C, Gerald WL, Rosail J (1996). Desmoid fibromatosis is a clonal process. Hum Pathol.

[B8] Lucas DR, Shroyer KR, McCarthy PJ (1997). Desmoid tumor is a clonal proliferation: PCR amplification of HUMARA for analysis of patterns of X-chromosome inactivation. Am J Surg Pathol.

[B9] Chansky HA, Trumble TE, Conrad EU, Wolff JF, Murray LW, Raskind WH (1999). Evidence for a polyclonal etiology of palmar fibromatosis. J Hand Surg [Am].

[B10] Lyon MF (1961). Gene action in the X-chromosome of the mouse (Mus musculus L). Nature.

[B11] Lyon MF (1998). X-chromosome inactivation: a repeat hypothesis. Cytogenet Cell Genet.

[B12] Allen RC, Zoghbi HY, Moseley AB, Rosenblatt HM, Belmont JW (1992). Methylation of HpaII and HhaI sites near the polymorphic CAG repeat in the human androgen-receptor gene correlates with X chromosome inactivation. Am J Hum Genet.

[B13] Saxena A, Alport EC, Custead S (1999). Molecular analysis of clonality of sporadic angiomyolipoma. J Pathol.

[B14] Rabkin CS, Janz S, Lash A (1997). Monoclonal origin of multicentric Kaposi's sarcoma lesions. N Engl J Med.

[B15] Rayan GM (1999). Clinical presentation and types of Dupuytren's disease. Hand Clin.

[B16] Moyer KE, Banducci DR, Graham WP, Ehrlich HP (2002). Dupuytren's disease: physiologic changes in nodule and cord fibroblasts through aging in vitro. Plast Reconstr Surg.

[B17] Hindman HB, Marty-Roix R, Tang JB, Jupiter JB, Simmons BP, Spector M (2003). Regulation of expression of [alpha]-smooth muscle actin in cells of Dupuytren's contracture. J Bone Joint Surg Br.

[B18] Baird KS, Crossan JF, Ralston SH (1993). Abnormal growth factor and cytokine expression in Dupuytren's contracture. J Clin Pathol.

[B19] Wever ID, Cin PD, Fletcher CDM, Mandahl N, Mertens F, Mitelman F, Rosai J, Rydholm A, Sciot R, Tallini G, Berghe HVD, Vanni R, Wille'n H (2000). Cytogenetic, Clinical, and Morphologic Correlations in 78 Cases of Fibromatosis: A Report from the CHAMP Study Group. Mod Pathol.

[B20] Wu Y, Basir Z, Kajdacsy-Balla A (2003). Resolution of clonal origins for endometriotic lesions using laser capture microdissection and the human androgen receptor (HUMARA) assay. Fertil and Steril.

[B21] Cong P, Raffeld M, Teruya-Feldstein J, Sorbara L, Pittaluga S, Jaffe ES (2002). In situ localization of follicular lymphoma: description and analysis by laser capture microdissection. Blood.

[B22] Kernek KM, Ulbright TM, Zhang S, Billings SD, Cummings OW, Henley JD, Michael H, Brunelli M, Martignoni G, Foster RS, Eble JN, Cheng L (2003). Identical allelic losses in mature teratoma and other histologic components of malignant mixed germ cell tumors of the testis. Am J Pathol.

[B23] Busque L, Mnio R, Mattioli J (1996). Nonrandom X-inactivation patterns in normal females : lyonization ratios vary with age. Blood.

[B24] Cheng L, Gu J, Ulbright TM, MacLennan GT, Sweeney CJ, Zhang S, Sanchez K, Koch MO, Eble JN (2002). Precise microdissection of human bladder carcinomas reveals divergent tumor subclones in the same tumor. Cancer.

[B25] Bamba M, Sugihara H, Okada K, Bamba T, Hattori T (1998). Clonal analysis of superficial depressed-type gastric carcinoma in humans. Cancer.

